# Excellent long-term outcome of renal transplantation in cystinosis patients

**DOI:** 10.1186/s13023-015-0307-9

**Published:** 2015-07-25

**Authors:** Camille Cohen, Marina Charbit, Bernadette Chadefaux-Vekemans, Magali Giral, Valérie Garrigue, Michèle Kessler, Corinne Antoine, Renaud Snanoudj, Patrick Niaudet, Henri Kreis, Christophe Legendre, Aude Servais

**Affiliations:** Service de Néphrologie-Transplantation, Hôpital Necker, 149 rue de Sèvres, 75015 Paris, France; Université Sorbonne Paris Cité, Paris, France; Service de Néphrologie pédiatrique, Hôpital Necker, APHP, Paris, France; Laboratoire de biochimie B, Hôpital Necker, Paris, France; Service de Néphrologie-Transplantation, CHU, Nantes, France; Service de Néphrologie-Transplantation, CHU, Montpellier, France; Service de Néphrologie-Transplantation, CHU, Nancy, France; Service de Néphrologie-Transplantation, hôpital Saint Louis, APHP, Paris, France

## Abstract

**Background:**

Cystinosis is a rare lysosomal disorder leading to end stage renal disease in more than 90 % of patients before 20 years of age. Data about safety and efficiency of renal transplantation in patients with cystinosis is scarce. We evaluated long-term outcomes of renal transplantation in adult patients with cystinosis.

**Methods:**

Data of renal transplantation (*n* = 31) in 30 adult patients with cystinosis in 5 French university transplant centers between 1980 and 2013 were retrospectively analyzed. A control cohort of 93 patients was matched for age, graft date, living/deceased donor status and transplant center.

**Results:**

Median age at transplantation was 20.4 years (7–36.5). At transplantation, all patients with cystinosis had corneal cystine deposits, 3 had diabetes and 7 had hypothyroidism. Graft survival was better in patients with cystinosis than in control patients (*p* = 0.013). Multivariate analysis confirmed that cystinosis was an independent protective factor for graft survival (Hazard Ratio (HR) 0.11; CI95 [0.02-0.61]). Specific complications of cystinosis occurred during follow up: diabetes mellitus (*n* = 4), hypothyroidism (*n* = 1), liver involvement (*n* = 1), neurologic involvement (*n* = 2). Proportion of post-transplant diabetes mellitus (PTDM) was not statistically different in cystinosis group compared to control group: 4 (13.0 %) compared to 5 (5.0 %), respectively (*p* = 0.25), with no differences regarding calcineurin inhibitors and steroids treatments during follow-up.

**Conclusions:**

Renal transplantation appears to be safe with excellent long-term outcomes in patients with cystinosis. These patients may receive standard immunosuppressive regimens with steroids and calcineurin inhibitors.

## Background

Cystinosis is a rare autosomal recessive lysosomal disorder with an estimated incidence of 1 per 100 000 to 200 000 live births[1]. The gene responsible for the disease, *CTNS*, was identified in 1998 [2]. A lot of mutations have since been described, and most lead to the complete loss of function of the cystinosin protein [3, 4]. Cystinosin is a protein required for the transport of free cystine from lysosome to cytoplasm. When cystinosin is deficient, free cystine accumulates in lysosome and forms crystals which are toxic for cells [5]. All organ involvement results from the accumulation of free cystine crystals leading to cell dysfunction [1, 5], an increase in autophagy [6],and activation of the inflammasome [7].

Clinical symptoms generally appear several months after birth and kidney involvement is the most serious clinical event, because it leads to end stage renal disease (ESRD) before the age of 20 in more than 90 % of patients [8, 9]. Nevertheless, other systemic manifestations may exist, such as hypothyroidism, diabetes mellitus, corneal crystal deposits, neurological (distal myopathy, cognitive disorder) and hepatic involvement (hepatosplenomegaly, nodular regenerative disorder). Corneal involvement may lead to corneal transplantation. Diagnosis is made by measuring leukocyte cystine content [10], by ophthalmologic examination which detects corneal cystine crystals, and by genetic testing.

Systemic treatment with a cystine-depleting agent, cysteamine, delays the time between diagnosis and the onset of ESRD, but is not sufficient to prevent ESRD [8]. Renal transplantation is the best therapeutic option for ESRD in young patients [11, 12], but few studies have investigated the safety and efficacy of kidney transplantation in cystinosis patients [13–15]. Furthermore, the long-term complications after kidney transplantation in adult cystinosis patients are unknown.

Here, we report the long-term outcome of kidney transplantation in adult cystinosis patients in a multicenter French cohort.

## Material and methods

Thirty adult cystinosis patients who received a kidney transplant at one of five French university centers were included in this retrospective study. A control cohort of 93 patients was established from the French database DIVAT (Données Informatisées et VAlidées en Transplantation) [16], with cystinosis as sole exclusion criteria. Each patient was matched with three controls for age, graft date, living/deceased donor status and center. After matching, each control was randomly selected in the list. Informed consent was obtained for all patients. The DIVAT database is approved by the French board Commission Nationale Informatique et Liberté (CNIL).

For deceased donor, extended criteria donor (ECD) was defined as: donor > 60 years old, or donor between 50 and 60 years old with at least hypertension, serum creatinine > 1.5 mg/dL or death of cerebrovascular cause [17]. Standard criteria donors were defined as donors without these ECD characteristics. Cellular- and antibody-mediated rejections (ABMR) were defined according to 2009 Banff criteria [18]. Antibody-mediated rejection was only assessed in biopsies performed after 2005. Delayed graft function was defined as a requirement for dialysis during the first week after transplantation. Estimated glomerular filtration rate (eGFR) was assessed by the MDRD formula [19].

Leukocyte cystine levels, reported as nmol hemi-cystine/mg protein were centrally measured (Dr Chadefaux-Vekemans) using liquid chromatography-tandem mass spectroscopy (API 3000LC/MS/MS; Applied Biosystems/MDS Sciex) with previously described methods [10]. Normal expected value is below 0.2 nmol hemi cystine/mg proteins.

Quantitative data are expressed as median (range). Statistical analysis was performed with GraphPad Prism 5 software (San Diego, CA, USA). The Student *t*-test was used to compare quantitative data, and the Fischer exact test was used to compare qualitative data. Kaplan-Meier curves with a log-rank test were performed for the survival analysis. A multivariate Cox model was secondarily used to identify factors associated with graft survival. A *p*-value < 0.05 was considered significant.

## Results

### Clinical and demographic data (Table 1)

**Table 1:** Clinical and demographic data of patients

	cystinosis (*n* = 31)	control (*n* = 93)	
Male / Female	16/15	59 / 34	
Age at ESRD (years)	18.8 (7.7-35.0)	19.8 (4.0-32.0)	*p* = 0.07
Age at transplantation (years)	20.4 (7.1-36.5)	21.8 (13.6-32.0) *	*p* = 0.03
Patient <19 yearsold (*n*=)	15 (48.4 %)	12 (12.9 %) *	*P* < 0.001
Time from dialysis to transplantation (years)	1.3 (0.12-8.0)	1.6 (0.1-19.0)	*p* = 0.1
Preemptive transplantation (*n*=)	9 (29.0 %)	12 (13.0 %)	*p* = 0.06
Transplantation rank	1 (*n* = 25); 2 (*n* = 6)	1 (*n* = 75); 2 (*n* = 17)	
BMI (kg/m^2^)	19.0 (14.3-28.7)	19.9 (14.0-25.0)	*p* = 0.4
Diabetesmellitus (*n*=)	3 (9.7 %)	2 (2 %)	*p* = 0.1
Initial graft data			
Living kidney donation (*n*=)	9 (29.0 %)	26 (28.6 %)	*p* = 1
Deceased donor age (years)	30.0 (0.6-64.0)	37.0 (10.0-62.0) *	*p* < 0.05
Extended criteria donor (*n*=)	1 (3.2 %)	3 (3.2 %)	*p* = 1
Delayed graft function (*n*=)	4 (13.0 %)	18 (19.0 %)	*p* = 0.6
Cold ischemia (hours)	17.5 (0.6-43.0)	17.5 (0.5-47.0)	*p* = 0.4
Donor specific antibody presence (*n*=)	4 (13.0 %)	13 (14.0 %)	*p* = 1
Induction treatment			
Steroids (*n*=)	30 (97.0 %)	93 (100 %)	*p* = 0.3
Mycophenolate mofetil (*n*=)	18 (58.0 %)	53 (57.0 %)	*p* = 1
Azathrioprine (*n*=)	13 (42.0 %)	15 (16.0 %)*	*p* 0.006
Ciclosporine A (*n*=)	14 (45.0 %)	53 (57.0 %)	*p* = 0.3
Tacrolimus (*n*=)	17 (55.0 %)	35 (37.6 %)	*p* = 0.1
OKT3 (*n*=)	0	5 (5.4 %)	*p* = 0.30
Thymoglobulin (*n*=)	18 (58.0 %)	53 (57.0 %)	*p* = 1
Basiliximab (*n*=)	13 (42.0 %)	35 (37.6 %)	*p* = 0.7
Follow up			
Duration of follow up (months)	144.1 (5.9-340.6)	72.0 (0.1-240.0) *	*p* < 0.0001
Age at last follow up (months)	32.7 (18.7-54.5)	29.7 (18.7-43.3) *	*p* = 0.01
ESRD at last follow up (*n*=)	6 (19.4 %)	29 (31.0 %)	*p* = 0.3
eGFR (MDRD) at 180 months (mL/min/1.73 m2)	53.7 (19.0-103.0)	47.4 (7.7-111.4)	*p* = 0.18
Maintenance treatment			
Stop of steroid during first year (*n*=)	3 (9.7 %)	15 (16.0 %)	*p* = 0.6
Mycophenolate mofetil (*n*=)	15 (48.3 %)	43 (46.2 %)	*p* = 0.84
Azathrioprine (*n*=)	8 (25.8 %)	25 (26.8 %)	*p* = 1
Ciclosporine A (*n*=)	15 (48.0 %)	41 (44.0 %)	*p* = 0.7
Tacrolimus (*n*=)	14 (45.0 %)	33 (35.0 %)	*p* = 0.4
Everolimus (*n*=)	1 (3.0 %)	2 (2.0 %)	*p* = 1
Graft complication			
Graft rejection (*n*=)	8 (26.0 %)	30 (32.0 %)	*p* = 0.7
Infections (*n*=)	14 (45.0 %)	56 (60.0 %)	*p* = 0.2
PTDM (*n*=)	4 (13.0 %)	5 (5.0 %)	*p* = 0.2
Stroke (*n*=)	1 (3.0 %)	0	*p* = 0.3
Myocardial infarction (*n*=)	0	1 (1.0 %)	*p* = 1
Death (*n*=)	1 (3.0 %)	2 (2.0 %)	*p* = 1

Continuous variables are shown as median ± SD.N, number; ESRD, end stage renal disease; BMI, body mass index; eGFR, estimated glomerular filtration rate; PTDM, post transplantation diabetes mellitus

We collected data from 31 renal transplantations performed in 30 patients with cystinosis between 1980 and 2013 and established a control cohort of 93 patients who underwent renal transplantation during the same period.

Median age at transplantation was respectively 20.4 (7.1–36.5) and 21.8 (13.6–32.0) years for patients with cystinosis and controls. Rate of adolescent patients younger than 19 at transplantation was significantly higher in cystinosis than in control group (48.4 % and 12.9 % respectively, *p* < 0.001).

Causes of ESRD in the control group were glomerular disease (*n* = 31, 33.3 %, including *n* = 7, 7.5 % of patients with focal segmental glomerulosclerosis), urinary tract malformations or vesico-ureteral reflux (*n* = 23, 24.7 %), renal genetic disease excluding cystinosis (*n* = 21, 22.6 %), hemolytic and uremic syndrome (*n* = 2, 2.2 %) or unknown cause (*n* = 16, 17.2 %).

Prior to transplantation, all patients with cystinosis had corneal cystine deposits, 3 of them (9.7 %) had diabetes mellitus, and 7 (22.6 %) had hypothyroidism.

All but one patient with available genetic data (20/30 patients) had loss of function mutations in the *CTNS* gene. The remaining patient had a mutation that partially impaired protein function (late-onset cystinosis).

### Kidney transplant characteristics (Table 1)

Nine (29.0 %) cystinosis patients and 26 (28.6 %) control patients received a kidney from a living donor. Median deceased donor age was respectively 30.0 (0.6–64) and 37.0 (10–62) years for patients with cystinosis and control patients (*p* < 0.05), respectively. Proportion of extended criteria donor was similar in both groups (3.2 % in both groups). Proportion of patients with donor specific anti-HLA antibody was similar in both groups.

Induction treatment was similar in both groups excepted for azathioprine (42.0 % and 16.0 % in cystinosis and control patients, respectively, *p* = 0.006)

The proportion of patients who underwent a preemptive transplantation tended to be higher in the cystinosis than in the control group: nine (29.0 %) vs12 (13.0 %) patients, respectively, *p* = 0.054).

### Long term outcomes and follow-up

During a median follow-up of 144.1 months (5.9–340.6) in the cystinosis group and 72.0 months (0.1–240.0) in the control group, 6 (19.4 %) and 29 (31.0 %) patients respectively developed ESRD. Median eGFR (MDRD) at 180 months tended to be higher in cystinosis group compared to control group: 53.7 (19.0–103.0) and 47.4 (7.7–111.4) ml/min/1.73 m^2^, respectively (*p* = 0.18).

At the end of follow up, patient survival was 97.0 and 98.0 % in the cystinosis and the control group, respectively.

**Fig. 1:**
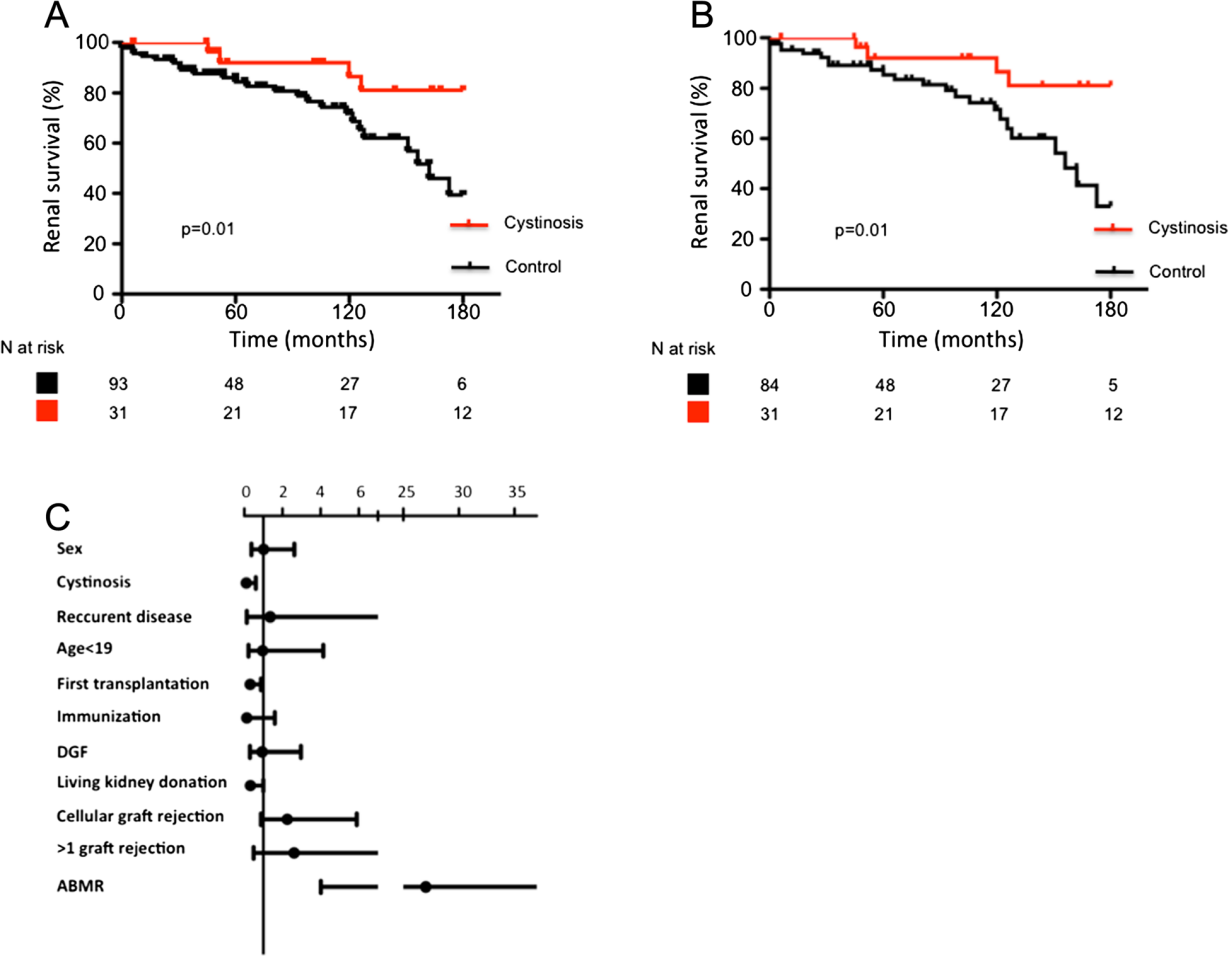
Renal survival **a**) Kaplan-Meier analysis of graft survival during follow up. Number of patients at risk is recapitulated in the table below the figure. **b**) Kaplan-Meier analysis of graft survival during follow up, excluding recurring diseases. Number of patients at risk is recapitulated in the table below the figure. **c**) Multivariate Cox model for associated factors with graft survival. Data are expressed as hazard ratio (spot) with 95 % confidence interval (bars)

Graft survival at 5 and 10 years was 92.0 and 86.5 % in cystinosis group, respectively, and 86.0 % and 72.0 % in control group. Graft survival was significantly better in cystinosis group than in control group (Figure 1a, *p* = 0.01), even when excluding patients with recurring diseases (Fig. 1b, *p* = 0.01).The proportion of patients experiencing graft rejection or infection was similar in both groups (Table 1). During follow up, biopsy-controlled graft rejection occurred in 8 (26 %) patients with cystinosis and 30 (32 %) patients in the control group (*p* = 0.7). Cellular rejection was involved in 62.5 % of rejections in the cystinosis group and 60 % of rejections in the control group. Antibody mediated rejection occurred in only three patients with cystinosis and six control patients. Resistance to treatment was similar in both groups (0 and 3 % respectively, *p* = 1).

Additionnal analysis performed after exclusion of the late-onset cystinosis patient did not modify significantly our results, especially regarding age at transplantation (19.5 years after exclusion, compared to 20.4) and graft survival.

The following parameters were selected for multivariate analysis of factors influencing graft survival (Table 2 and Fig. 1c): sex, cystinosis, possibly recurrent disease, age < 19, immunization, antibody mediated rejection, cellular graft rejection, more than one episode of graft rejection, first transplantation, delayed graft function, living kidney donation. Cystinosis was confirmed as a protective factor for graft survival (HR = 0.11; CI95 [0.02–0.61]), as well as first transplantation (HR = 0.31; CI95 [0.11–0.87]) and living kidney donation (HR = 0.32; CI95 [0.10–1.00]). Antibody mediated rejection was the sole pejorative factor associated with graft survival (HR = 27.03; CI95 [4.02–181.96]).

**Table 2:** Multivariate Cox model for associated factors with graft survival

	HR	Std. Err.	z	*P* > |z|	[95 % CI]
Sex	1.01	0.49	0.01	1,00	[0.38-2.63]
Cystinosis	0.11	0.09	−2.53	0.01	[0.02-0.61]
Reccurent disease	1.36	1.63	0.25	0.80	[0.13-14.27]
Age < 19	0.96	0.72	−0.05	0.96	[0.22-4.15]
First transplantation	0.31	0.16	−2.23	0.03	[0.11-0.87]
Immunization	0.14	0.17	−1.58	0.11	[0.01-1.60]
DGF	0.94	0.55	−0.1	0.92	[0.30-2.97]
Living kidney donation	0.32	0.19	−1.95	0.05	[0.10-1.00]
Cellular graft rejection	2.26	1.11	1.67	0.09	[0.87-5.90]
>1 graft rejection	2.61	2.21	1.13	0.26	[0.49-13.77]
ABMR	27.03	26.30	3.39	0.001	[4.02-181.96]

ABMR: antibody-mediated rejection, DGF: delayed graft function, HR: hazard ratio, Std. Err., standard error

**Fig. 2:**
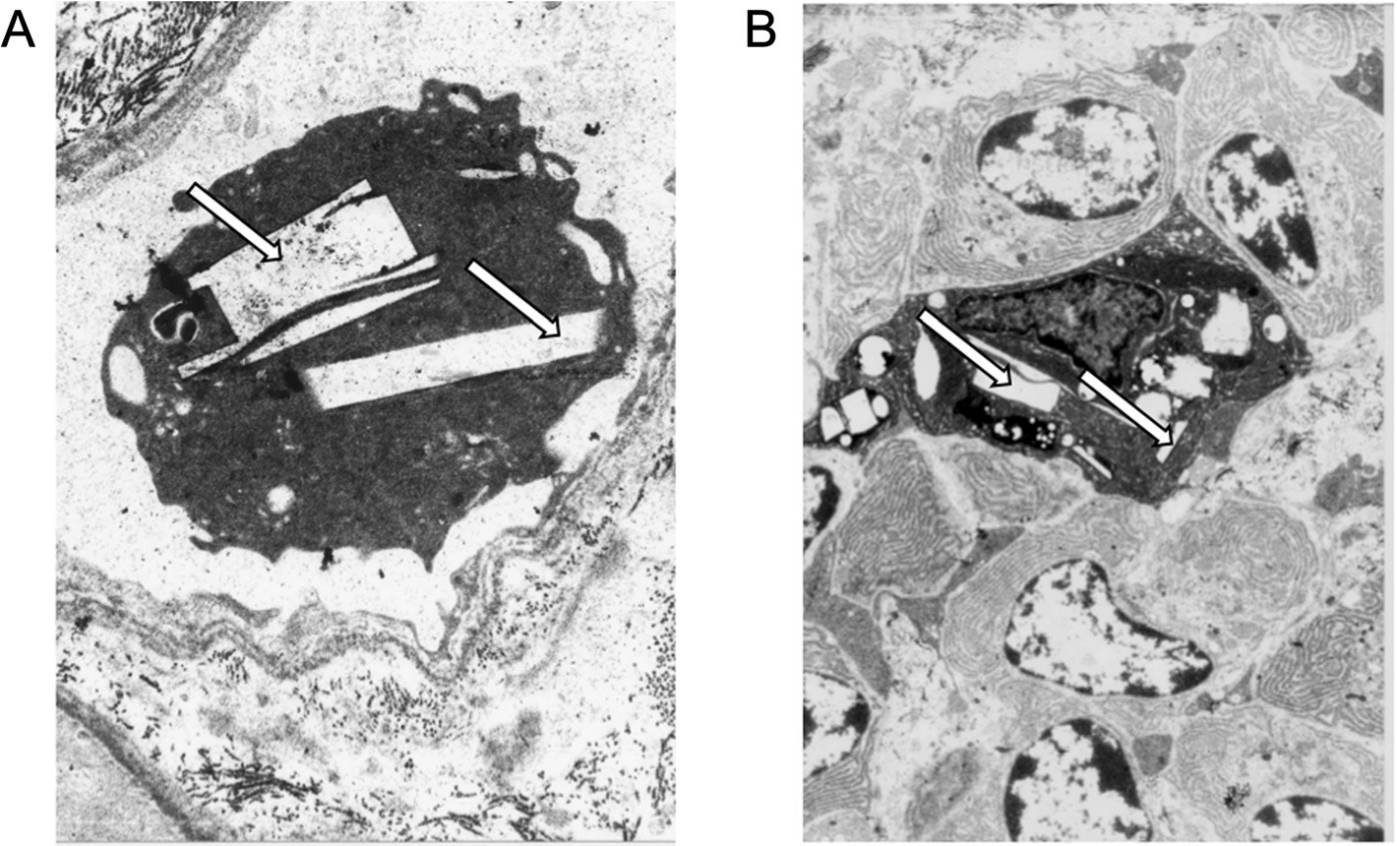
Kidney transplant biopsy showing cystine crystal (arrows) into recipient mononuclear cells. **a**). Intracapillary circulating lymphocyte with cystine crystals (arrows). Electron microscopy, magnification x5000. **b**). Cystine crystals (arrows) in a macrophage infiltrating the mesangium. Electron microscopy, uranyl lead staining, magnification x2400. Photo Dr MC Gubler, and Dr GS Spear

A protocol biopsy was performed one year after transplantation in 13 patients with cystinosis and cystine crystals were observed in the renal biopsy from only one patient (Fig. 2). However, this finding did not negatively influence prognosis because the patient had a functional graft 22 years after transplantation (serum creatinine level 182 μM).

Proportion of post-transplant diabetes mellitus (PTDM) was not statistically different in cystinosis group compared to control group: 4 (13.0 %) compared to 5 (5.0 %), respectively (*p* = 0.25). The median time to diabetes onset was 78 months (3–180) in the cystinosis group, compared to 3 months (0.1–187) in the control group. The prevalence of vascular complications was also similar in the two groups: one patient with cystinosis had a stroke 3 years after transplantation, whereas one control patient had a myocardial infarction 2 years after transplantation.

Maintenance treatment was similar in both groups: in particular, calcineurin inhibitors were given to 29 (93.0 %) cystinosis patients and 74 control patients (79.0 %) (*p* = 0.1), with similar proportion of patients treated by ciclosporine A and tacrolimus (Table 1). Steroids were discontinued during the first year in three (9.7 %) cystinosis patients and15 control patients (16.0 %) (*p* = 0.6).

### Complications of cystinosis after renal transplantation

Complications associated with cystinosis occurred during follow up, including diabetes mellitus (*n* = 4), hypothyroidism (*n* = 1), hepatic complications (*n* = 1) and neurological involvement (*n* = 2). Neurological complications consisted in progressive distal myopathy in two patients, leading to interdigital amyotrophy. One of these patients also had restrictive pulmonary disease and swallowing dysfunction. The other patient had pseudo-ischemic stroke lesions on magnetic resonance imaging (MRI). He presented 3 years after transplantation with behavioral disorder without any motor or sensitive deficiency. MRI revealed T2 hyperintensity in the middle cerebral artery territory, but MR angiography did not showed any evidence of vessels stenosis. Carotide artery doppler was normal. In this context, cystinosis-related encephalopathy was suspected. The patient with hepatic involvement presented with cholestasis.. Ultrasound liver examination found moderate portal hypertension, without bile duct dilation or mass. Antibodies against smooth muscle, actin, mitochondria liver kidney molecule (LKM), and soluble liver antigen were negative. There was no infection for HBV, HCV or HIV. There was no iron overload. No liver biopsy was performed but cholestasis was probably related to cystinosis.

Corneal transplantation was performed in two patients during follow up. One patient died after 51 months of follow-up, because of a car accident.

The median leukocyte cystine content was 1.4 nmol hemicystine/mg protein (0.4–4.7) at day 0, and 1.9 (0.5–10.0) during follow up (objective below 1). All patients who developed cystinosis complications had cystine leukocyte content above 2 at the time of the complication.

One patient developed cavum post transplantation lymphoproliferative disorder, successfully treated with chemotherapy. One patient experienced ankle melanoma successfully treated with surgery.

## Discussion

We report, to our knowledge, one of the largest cohorts of adult patients with cystinosis undergoing renal transplantation with long-term follow up. Cysteamine therapy has greatly improved the prognosis of cystinosis, and delayed the onset of end-stage renal disease, but is not sufficient to avoid dialysis [8, 15, 20]. Renal transplantation is the best therapeutic option for ESRD and improves both survival rates and quality of life. However, few studies have examined the outcome of renal transplantation in cystinosis patients, and there is no data about long-term outcomes of renal transplantation at the era of cysteamine therapy. We demonstrate here a better graft survival compared to matched controls and show that cystinosis is an independent protective factor for graft survival. We also describe some late complications of the disease.

In pediatric cohorts, the outcome of kidney transplantation in patients with cystinosisis generally better than that for other patients undergoing transplantation [15, 21]. Indeed, results from the database of the North American Pediatric Renal Transplant Cooperative Study suggest that the outcome of renal transplantation is favorable in patients with a primary diagnosis of cystinosis, whereas a diagnosis of congenital nephrotic syndrome or focal segmental glomerulosclerosis associated with a high risk of graft failure, as expected [21]. More recently, data collected between 1979 and 2008 by the European Society for Paediatric Nephrology (ESPN)/European Renal Association and European Dialysis and Transplant Association (ERA/EDTA) registry showed that 5-year graft survival was better in 245 cystinosis patients compared to 490 control children undergoing kidney transplantation for another cause (94.0 % versus 84.0 %, respectively) [15]. Similarly, the Australian and New Zealand (ANZDATA) registry showed encouraging results: the 10-year death-censored survival of first transplant recipients with cystinosis (*n* = 44) was 86% at the era of cysteamine therapy [22]. Here, we report similar rate of graft survival at 5 and 10 years among both patients with cystinosis and control patients (92 % and 86 % at 5 years, 86.5 and 72 % at 10 years, respectively). Furthermore, we show that graft survival is better in patients with cystinosis compared to controls with respectively 180 months graft survival of 81 % and 39 % (*p* = 0.01) and 53 % of patients with cystinosis have a functional graft at 340 months of follow up. We confirmed, by multivariate analysis, that cystinosis is an independent protective factor for graft survival.

Interestingly, preliminary unpublished data suggest that the mTORC1 pathway is down-regulated in cystinosis patients, which may explain the very good long-term outcome of renal transplantation in these individuals, and the previously suggested better immune tolerance of patient with cystinosis [23]. In our cohort, deceased donors were older in the control than in the cystinosis group (37 vs. 30 years, respectively, *p* < 0.05), but this small difference is unlikely to explain the large difference in long-term graft survival. Actually, the proportion of well-defined standard criteria donor was strictly similar in both groups. We compared patients with cystinosis with age-matched control patients, including the usual renal diseases observed at this age: 9 control patients (9.7 %) had a renal disease with potential recurrence in the transplant: 7 had primary focal and segmental glomeruslosclerosis and 2 had hemolytic and uremic syndrome. These diseases are known to have bad prognosis after kidney transplantation, and could participate in the long-term difference in graft survival we observed. But, after exclusion of these 9 patients, we confirmed that graft survival was better in patients with cystinosis than in control patients. Furthermore, multivariate analysis including recurring disease in the analysis, confirmed that cystinosis was an independent protective factor for graft survival.

We confirmed that ABMR is a pejorative factor. However, surprisingly, immunization tended to be a protective factor, but statistical significance was not reached. These data are probably not clinically relevant, since our cohort is too small.

We also found that the rate of patient survival was excellent (97.0 % at 10 years), whereas prognosis in the ANZDATA study was poorer, with 88 % survival at 10 years in patients treated with cysteamine [22]. However, in these two cohorts, at the era of cysteamine, none of the deaths was related to cystinosis or chronic kidney disease. Thus we can suppose that this difference is not relevant regarding to the specific outcome of cystinosis after transplantation. In the opposite, in the early group (no cysteamine) of the ANZDATA study, the majority of deaths were cystinosis or chronic kidney disease related.

One of the limits of our study is that patients with cystinosis have been followed longer than controls (144 and 72 months, respectively). Indeed, because cystinosis is a rare disease, patients may be followed longer in tertiary centers, introducing bias into the very long-term data. However, a longer follow-up, as well as a more important proportion of adolescent patients in cystinosis group, should drag survival data down. Indeed, it has been shown that adolescent patients have lower rate of graft survival compared to other age groups [24]. Furthermore, a longer follow up in the cystinosis group may have overestimated the rate of long term post transplantation complication compared to control patients. Interestingly, in our study, median age at the time of renal replacement therapy was higher than that reported in previous studies, with values ranging between 10.1 and 12.8 years [15, 22]. Van Stralen et al. .refer to their findings as ‘disappointing’, because the expected age at ESRD onset is above 18 years among patients treated with cysteamine. Nevertheless, a recent analysis of the ERA/EDTA registry showed that 17/26 incident patients with cystinosis-related ESRD were older than 20 years [25].

Proximal tubular disease does not occur in the transplanted kidney because cystine lysosomal efflux transport system is functional in the engrafted kidney. However, retention of a native kidney can result in the persistence of renal tubular Fanconi syndrome. Interestingly, one year after transplantation, one patient developed cystine crystals in the transplanted kidney, which were diagnosed on protocol biopsy. Nonetheless, the outcome of renal transplantation in this patient was excellent, and serum creatinine was 186 μM more than 20 years after transplantation. Indeed, protocol biopsies have shown that cystine crystals may be deposited within the interstitial tissue and less frequently within glomeruli without any clinical or biological manifestations. These crystals are present in the host’s own mononuclear cells which have subsequently infiltrated the transplant [26, 27].

Renal transplantation does not correct the systemic metabolic defect of cystinosis and cystine continues to accumulate in most organs and will lead to late systemic complications. In particular, the cystinotic process is known to gradually alter beta-cell function over the years [28]. Because pancreatic insufficiency may trigger post-transplant diabetes mellitus during glucocorticoid-based immunosuppression [29], the use of steroid-free immunosuppressive regimens has also been proposed [30]. In our cohort, patients with cystinosis received the same immunosuppressive regimens than other patients, particularly the proportion of patients treated with tacrolimus, which is involved in the development of post-transplant diabetes mellitus, was similar in both groups. Numerically more cystinosis patients developed diabetes compared to controls but it was not statistically significant. The median time to diabetes onset post transplantation was 78 months, confirming that, in patients with cystinosis, diabetes can develop a long time after transplantation, and doesn’t seem to be related to immunosuppressive regimen. The use of a cystine-depleting agent (cysteamine) by all patients during follow-up may explain this favorable outcome, because this agent probably limits the risk of cystinosis-related diabetes. Thus, immunosuppressive maintenance treatment regimens should be equivalent between patients with or without cystinosis, because the potential risk of diabetes after transplantation is not sufficient to necessitate specific treatment in patients with cystinosis.

Two patients developed neurological complications of cystinosis during follow up, with myopathy leading in one patient to a restrictive pattern of chronic respiratory failure. These neurological complications were first described many years ago, but their incidence after transplantation and the effectiveness of cysteamine therapy in preventing them are poorly documented [9, 30].

All cystinosis-related complications during follow up occurred in patients with high cystine leukocyte content, showing that maintaining cysteamine treatment is critical for the management of these patients. We recommend regular monitoring of cysteine leukocyte content and treatment with cysteamine must be continued to maintain cystine levels below 1 nmol hemicystin/mg of protein.

## Conclusion

In summary, we report that the long-term outcome of cystinosis patients undergoing renal transplantation is excellent. Furthermore, our findings show that renal transplant recipients with cystinosis have a better long-term outcome than other renal transplant recipients, and that cystinosis is an independent protective factor for graft survival. The moderate risk of post-transplant diabetes mellitus does not necessitate the use of specific immunosuppressive agents in cystinosis patients. Our work supports recently published consensus guidelines for the diagnosis and treatment of cystinosis [31]. Although cystinosis is a rare disease, all physicians involved in renal transplantation should be aware of the treatment regimens and specific complications associated with this disease.

## Informed consent

All procedures followed were in accordance with the ethical standards of the responsible committee on human experimentation and with the Helsinki Declaration of 1975, as revised in 2000. Informed consent was obtained from all patients for being included in the study. The clinical and research activities being reported are consistent with the Principles of the Declaration of Istanbul as outlined in the 'Declaration of Istanbul on Organ Trafficking and Transplant Tourism’.

## Abbreviations

ABMR: Antibody mediated rejection
CNIL: Commission informatique et liberté
CTNS: Cystinosin
DGF: Delayed graft function
DIVAT: Données Informatisées et VAlidées en Transplantation
ECD: Extended criteria donors
eGFR: estimated Glomerular filtration rate
ESRD: End stage renal disease
MDRD: Modification of diet in renal disease
PTDM: Post-transplant diabetes mellitus
SCD: Standard criteria donors
